# Joint Effect of Beer, Spirits Intake, and Excess Adiposity on Hyperuricemia Among Chinese Male Adults: Evidence From the China National Health Survey

**DOI:** 10.3389/fnut.2022.806751

**Published:** 2022-02-22

**Authors:** Huijing He, Li Pan, Xiaolan Ren, Dingming Wang, Jianwei Du, Ze Cui, Jingbo Zhao, Hailing Wang, Xianghua Wang, Feng Liu, Lize Pa, Xia Peng, Chengdong Yu, Ye Wang, Guangliang Shan

**Affiliations:** ^1^Department of Epidemiology and Statistics, Institute of Basic Medical Sciences, Chinese Academy of Medical Sciences and School of Basic Medicine, Peking Union Medical College, Beijing, China; ^2^Department of Chronic and Noncommunicable Disease Prevention and Control, Gansu Provincial Center for Disease Control and Prevention, Lanzhou, China; ^3^Department of Chronic and Noncommunicable Disease Prevention and Control, Guizhou Provincial Center for Disease Control and Prevention, Guiyang, China; ^4^Department of Chronic and Noncommunicable Disease Prevention and Control, Hainan Provincial Center for Disease Control and Prevention, Haikou, China; ^5^Department of Chronic and Noncommunicable Disease Prevention and Control, Hebei Provincial Center for Disease Control and Prevention, Shijiazhuang, China; ^6^Department of Epidemiology and Statistics, School of Public Health, Harbin Medical University, Harbin, China; ^7^Department of Chronic and Noncommunicable Disease Prevention and Control, Inner Mongolia Autonomous Region Center for Disease Control and Prevention, Hohhot, China; ^8^Integrated Office, Institute of Biomedical Engineering, Chinese Academy of Medical Sciences, Peking Union Medical College, Tianjin, China; ^9^Department of Chronic and Noncommunicable Disease Prevention and Control, Shaanxi Provincial Center for Disease Control and Prevention, Xi'an, China; ^10^Department of Chronic and Noncommunicable Disease Prevention and Control, Xinjiang Uyghur Autonomous Region Center for Disease Control and Prevention, Urumqi, China; ^11^Department of Chronic and Noncommunicable Disease Prevention and Control, Yunnan Provincial Center for Disease Control and Prevention, Kunming, China

**Keywords:** interaction effect, hyperuricemia, excess adiposity, alcohol intake, modifiable risk factor

## Abstract

Alcohol intake and excess adiposity are associated with serum uric acid (SUA), but their interaction effect on hyperuricemia (HUA) remains unclear. Using data from the China National Health Survey (CNHS) (2012–2017), we analyzed the additive interaction of beer, spirits intake, excess adiposity [measured by body mass index (BMI), body fat percentage (BFP), and visceral fat index (VFI)] with HUA among male participants aged 20–80 from mainland China. The relative excess risk due to interaction (RERI), the attributable proportion due to interaction (AP), and the synergy index (SI) were calculated to assess the interaction effect on the additive scale. Both RERI and AP larger than 0 and SI larger than 1 indicate a positive additive interaction. Among 12,592 male participants, the mean SUA level was 367.1 ± 85.5 μmol/L and 24.1% were HUA. Overweight/obese men who were presently drinking spirits had an odds ratio (OR) of 3.20 (95%CI: 2.71–3.79) than the never drink group, with RERI, AP, and SI of 0.45 (95%CI: 0.08–0.81), 0.14 (95%CI: 0.03–0.25), and 1.25 (95%CI: 1.02–1.54), respectively. However, although combined exposures on beer intake and excess adiposity had the highest OR compared with no beer intake and nonobese participants, there was no additive interaction, with RERI, AP, and SI in the overweight/obesity and the beer intake group of 0.58 (−0.41–1.57), 0.17 (−0.08–0.41), and 1.30 (0.85–1.97), respectively. Other excess adiposity indexes revealed similar estimates. Our findings suggested that the exposures of both excess adiposity and alcohol drink could result in an additive interaction effect on HUA: the combined risk of excess adiposity with spirits intake but not with beer was greater than the sum of the effects among Chinese male adults.

## Introduction

Hyperuricemia (HUA) is a causal component necessary for the development of gout (1) and is associated with multiple cardiometabolic diseases (1, 2). The prevalence of HUA in the US population was over 21% based on the data from the US National Health and Nutrition Examination Survey (NHANES) 2007–2008 (3). The data from the China National Health Survey (CNHS) showed that the prevalence of HUA was as high as 25.1% in men and 15.9% in women in mainland China (4).

Body composition and alcohol intake are known determinants of the serum uric acid (SUA) level (5–8). Based on NHANES-III, body mass index (BMI) and alcohol use could be used to individually account for a notable proportion of HUA cases (5). Our previous work revealed that, in men, the prevalence of overweight/obesity and current alcohol drinking was high, that is, over 40 and 65%, respectively. The proportions of HUA cases that could be attributed to overweight/obesity and alcohol consumption were 20.6 and 12.8%, respectively (4). It is well known that excess adiposity and alcohol consumption are the most essential modifiable risk factors for cardiometabolic diseases, and previous studies have explored their interaction role toward metabolic health (9, 10). However, whether the combined effect of hazardous alcohol use and excess adiposity increase the risk of HUA beyond the sum of their individual effects, i.e., their interaction effect on HUA, is still unclear. Shiraishi and Une explored the interaction of obesity and drinking on HUA in Japanese male office workers (7), but they did not distinguish the alcohol beverage kinds. Previous studies have shown that beer and spirits intake, but not wine, was associated with the SUA level (8, 11, 12). Furthermore, for assessing the public health importance, measuring interaction on the additive scale has been acknowledged as the most appropriate method in the epidemiological community. The additive scale indicates whether the effect of a risk factor would be greater in one subpopulation than in another and is thus useful in targeting intervention to certain subgroups if resources are limited (13). However, most studies estimating the risk difference are often reported using odds ratios (ORs) given that logistic regression is used for covariate adjustment and then the interaction is often not reported on the additive scale (7). The measurement of interaction on the additive scale (14), such as the relative excess risk due to interaction (RERI), the attributable proportion due to interaction (AP), and synergy index (SI) can be used to assess whether there is a synergism between excess body fat and alcohol use. As previous studies have indicated the causal effect of excess body fat and alcohol use on HUA (6, 15), RERI > 0 can imply such synergism (16). AP was intended to capture the proportion of the disease in the doubly exposed group that is due to the interaction between the two exposures (17).

Our previous work indicated that over 65% of men were obese, whereas 32% were women due to their much less alcohol drinking behaviors as well as lower alcohol intake dose than men (4). The combined effect of excess adiposity and alcohol consumption, thereby, may be more essential for the male population from the public health prospective. Therefore, using data from the CNHS, we aimed to estimate the joint effect of excess adiposity and different kinds of alcohol use on HUA among male adults in mainland China.

## Methods

### Study Population

Using a multistage, stratified sampling method, from 2012 to 2017, the CNHS selected a representative sample of the Chinese population in mainland China. More details are available from our previous publication (18). In brief, the CNHS collected data on demographic and socioeconomic information, lifestyle factors, anthropometric measures, laboratory tests, and clinical profiles from 11 provinces in mainland China. Only individuals aged 20–80 and who was lived in a local residence for at least 1 year were eligible to participate. Pregnant women, soldiers on active service, severe mental disease patients, or disabled people were excluded. Our analysis was limited to male adults who underwent biochemical tests and physical examination.

The study has been carried out in accordance with the Declaration of Helsinki. Ethical approval was obtained from the Bioethical Committee of the Institute of Basic Medical Sciences, Chinese Academy of Medical Sciences (No. 029-2013). All participants provided written informed consent before the survey.

### SUA Measurement and Definition of HUA

Venous blood samples were drawn after an overnight fast. Separated plasma or serum was frozen in aliquots and stored at −80°C until thawed for the first time for the analyses. The SUA level was measured by oxidization with the specific enzyme uricase on a Chemistry Analyzer (ROCHE Cobas8000C701, USA). HUA was defined as SUA > 420 μmol/L (7 mg/dl) based on the Guideline for primary care of gout and HUA in China (version 2019) (19).

### Assessment of Excess Adiposity and Alcohol Use

Height was measured to the nearest 0.1 cm using a fixed stadiometer. Weight, body fat percentage (BFP), and visceral fat index (VFI) were measured by using a body composition analyzer (TANITA BC-420, Japan), with the accuracy on a decimal level. BMI was calculated in kg/m^2^.

Based on the World Health Organization (WHO)'s definition of overweight and obesity, underweight was defined as BMI <18.5 kg/m^2^, normal weight was BMI ≥18.5 kg/m^2^ but <25 kg/m^2^, overweight was BMI ≥ 25 kg/m^2^ but <30 kg/m^2^, and obesity was BMI ≥ 30 kg/m^2^(20).

Information on alcohol use was collected by a self-report questionnaire interview. Alcohol use status was classified into three groups: never drinking, quit drinking, and current drinking. Current drinking was defined as the consumption of at least 30 g of alcohol and lasted for at least 6 months. In this study, participants who were drinking or quit drinking <6 months were classified into the “alcohol drinking in the past 1 year” group. Alcohol risk level was classified into three groups based on the WHO's guideline: low (1–40 g/day), medium (41–60 g/day), and high (>60 g/day) (21). We also collected information on the self-report consumption of beer, spirits, and wine through interview. We did not consider wine drinking in the final analyses due to its low consumption.

### Measurement of Other Covariates

Demographic and socioeconomic factors (age, living in urban or rural areas, education level, and personal annual income) were collected by a face-to-face standardized questionnaire interview. Education levels were grouped into three categories: illiterate or elementary school, high school, and college or over. Personal annual income was classified into four groups: <10,000, 10,000–29,999, 30,000–49,999, and not <50,000 CHY/year (1 CHY = 0.16 USD). Current tobacco use was defined as smoking at least one cigarette per day for at least 6 months. Former tobacco use was defined as having quit tobacco use for more than 6 months preceding the survey (18). The estimated glomerular filtration rate (eGFR, ml/min per 1.73 m^2^) was calculated according to the Modification of Diet in Renal Disease equation for Chinese (c-MDRD) (22). The formula for eGFR calculation is:


(1) $$\begin{array}{l} eGFR\;=\;175\times Scr^{-1.234}\times age^{-0.179}\times 0.742\left(if\;female\right) \end{array}$$

Scr is serum creatinine in mg/dl.

### Statistical Analyses

The final analytical samples were restricted to subjects without missing value of major factors of interest (age, alcohol drinking, and SUA level) and eGFR ≥ 60 ml/min per 1.73 m^2^ (to exclude possible kidney disorders (23)), no self-report of gout, or the use of diuretic medication. The original data of CNHS contain 53,895 participants with a multiethnic background. After excluding minority ethnic population (*n* = 21,588), missing values on SUA (*n* = 2,608), people did not live in the current residence (*n* = 55), eGFR <60 ml/min per 1.73 m^2^ (*n* = 506), female (*n* = 19,045), missing values on alcohol drinking (*n* = 109), the final sample included 12,592 participants.

Continuous variables were presented as means with SDs (if Gaussian distribution satisfied) or median with interquartile range (IQR), and categorical data as frequencies and percentages (%). The risk factors of interest were categorized as follows: BMI (≤ 25, 25–29, ≥30), BFP (≤ 25%, >25%), VFI (<15, ≥15), spirits intake level (classified into four groups, never, low, moderate, and high based on the conversion of daily alcohol intake and the same with the overall alcohol risk assessment), beer intake level (*n*ever, low: no more than 20 bottles 1 year, moderate: no more than 40 bottles 1 year, and high: more than 40 bottles 1 year).

RERI, AP, and SI were used to assess the additive interaction.

Relative excess risk due to interaction is defined as


(2) $$\begin{array}{l} RERI=RR_{11}-RR_{10}-RR_{01}+1 \end{array}$$

where RR_*ab*_ is the relative risk (RR) in the group with exposures *a* and *b* (1 = exposed, 0 = unexposed) as compared with the doubly unexposed group. A RERI equals 0 implying no additive interaction and >0 indicates a positive interaction (24). In this study, RR is replaced with OR yielded by the logistic regression model.

The attributable proportion due to interaction is defined as


(3) $$\begin{array}{l} AP=\left(RR_{11}-RR_{10}-RR_{01}+1\right)/RR_{11}=RERI/RR_{11} \end{array}$$

The synergy index is defined as


(4) $$\begin{array}{l} SI=\left(RR_{11}-1\right)/\left(\left(RR_{01}-1\right)+\left(RR_{10}-1\right)\right) \end{array}$$

If the 95% CIs of AP and SI did not include 0 and 1, respectively, then the additive interaction is presented. RERI, AP, and SI were estimated using the regression coefficients and a covariance matrix obtained from the logistic regression models (25, 26). The delta method was used to calculate the 95% CIs of RERI, AP, and SI (25, 27). For a sufficient power of joint effect estimation and ease of interpretation, alcohol use, and excess adiposity, were subsequently dichotomously grouped in the additive interaction analyses.

Comparisons of prevalence among groups were adjusted for potential confounders using logistic regression models (28). Multilevel linear regression models were used to obtain estimates between risk factors of interest and the SUA level. Survey logistic regression models were used to estimate the effect of excess adiposity and alcohol use on HUA.

We used SAS software version 9.4 to perform all the analyses. All *P*-values were two-sided, and α = 0.05.

## Results

### Subject Characteristics

A total of 12,592 male adults were included in the final sample. The mean age of the study population was 49.0 ± 13.5 years. Most participants were from urban areas (62.45%), had a high school educational level (51.45%), and had a moderate personal annual income (10,000–49,999 CHY, 62.89%). The mean SUA level was 367.1 ± 85.5 μmol/L and 24.1% were HUA. The prevalence and distribution of risk factor categories are presented in Table 1.

**Table 1:** Sociodemographic and health characteristics of male participants in mainland China.

**Characteristics**	**Overall (*n =* 12,592)**		**Non-HUA (*n =* 9,563)**		**HUA (*n* = 3,029)**		***P***
Age (years) (mean, SD)	48.98	13.46	49.62	13.43	46.96	13.36	<0.001
Age groups (*n*, %)							
20–	1296	10.29	920	9.62	376	12.41	<0.001
30–	2039	16.19	1440	15.06	599	19.78	
40–	3206	25.46	2406	25.16	800	26.41	
50–	3152	25.03	2451	25.63	701	23.14	
60–	2136	16.96	1723	18.02	413	13.63	
70–80	763	6.06	623	6.51	140	4.62	
Residential areas (*n*, %)							
Urban	7,864	62.45	5,702	59.65	2,162	71.38	<0.001
Rural	4,724	37.52	3,857	40.35	867	28.62	
Educational level (*n*, %)							
Illiterate or elementary school	2,273	17.70	1,851	19.36	422	13.93	<0.001
High school	6,452	51.45	4,968	51.95	1,484	48.99	
College	3,852	30.85	2,735	28.6	1,117	36.88	
Personal year income (CHY) (*n*, %)							
<10,000	2,166	17.2	1,822	19.05	344	11.36	<0.001
10,000-	3,650	28.99	2,835	29.65	815	26.91	
30,000-	4,269	33.9	3,154	32.98	1,115	36.81	
≥50,000	2,348	18.65	1,623	16.97	725	23.94	
BMI (kg/m^2^) (mean, SD)	24.41	3.53	23.92	3.40	25.93	3.50	<0.001
BMI category (*n*, %)							
Under/normal weight	7,172	56.96	5,983	62.56	1,189	39.25	<0.001
Overweight	4,573	36.32	3,103	32.45	1,470	48.53	
Obesity	724	5.75	385	4.03	339	11.19	
Body fat percentage (%) (mean, SD)	21.31	5.48	20.55	5.49	23.82	4.66	<0.001
Visceral fat index (mean, SD)	11.26	4.26	10.83	4.35	12.62	3.64	<0.001
eGFR (ml/min/1.73m^2^) (median, IQR)	97.47	24.90	99.51	24.47	90.37	23.51	<0.001
Alcohol drink in the past 1 year (*n*, %)							
No	4,358	34.61	3,545	37.07	813	26.84	<0.001
Yes	8,234	65.39	6,018	62.93	2,216	73.16	
Never smoke (*n*, %)	3,935	31.25	2,944	30.79	991	32.72	0.043
Ever smoke (*n*, %)	8,655	68.73	6,619	69.21	2,036	67.22	
Beer intake (*n*, %)							
Never	7,462	59.26	5,812	60.78	1,650	54.47	<0.001
No more than 20 bottles 1 year	3,370	26.76	2,547	26.63	823	27.17	
No more than 40 bottles 1 year	1,017	8.08	727	7.60	290	9.57	
More than 40 bottles 1 year	741	5.88	477	4.99	264	8.72	
Spirits intake level^#^ (*n*, %)							
Never	4,059	32.23	3,297	34.48	762	25.16	<0.001
Low	5,891	46.78	4,330	45.28	1,561	51.54	
Moderate	634	5.03	446	4.66	188	6.21	
High	1,170	9.29	832	8.70	338	11.16	
Serum creatinine (μmol/L) (mean, SD)	82.00	12.00	80.38	11.48	87.12	12.20	<0.001

# *The grouping criteria of spirits intake level, low: daily alcohol intake <40 g/day, moderate: 41–60 g/day, high: >60 g/day*.

*HUA, hyperuricemia; BMI, body mass index; eGFR, estimated glomerular filtration rate; 1CHY, 0.16USD; SD, standard deviation; IQR, Interquartile range*.

### Effect of Body Composition and Alcohol Use on SUA and HUA

The SUA level adjusted for covariates among body composition and alcohol use groups is shown in Table 2.

**Table 2:** Adjusted serum uric acid (SUA) levels in stratified groups and the effect of excess adiposity, beer, and spirits intake on SUA (*n* = 12,592).

**Model 1**				**Model 2**			
	**SUA (μmol/L, adjusted mean, 95%CI)**	**B (95%CI)**	**P**		**SUA (μmol/L, adjusted mean, 95%CI)**	**B (95%CI)**	**P**
**BMI category**				**BMI category**			
Under/normal weight	356.74(354.24–359.25)	Ref	NA	Under/normal weight	360.89(357.97–363.81)	Ref	NA
Overweight	390.77(387.94–393.60)	34.03(31.23–36.83)	<0.001	Overweight	394.91(391.68–398.14)	34.02(31.07–36.97)	<0.001
Obesity	421.11(415.27–426.94)	64.36(56.98–71.75)	<0.001	Obesity	424.07(417.95–430.19)	63.18(55.90–70.46)	<0.001
**BFP (%)**				**BFP (%)**			
≤ 25	355.41(352.84–357.98)	Ref	NA	≤ 25	359.34(356.36–362.33)	Ref	NA
>25	394.29(391.58–397.00)	38.88(36.43–41.32)	<0.001	>25	398.24(395.12–401.36)	38.90(36.46–41.33)	<0.001
**VFI**				**VFI**			
<15	365.48(363.08–367.88)	Ref	NA	<15	369.54(366.71–372.38)	Ref	NA
≥15	399.80(396.24–403.37)	34.32(30.39–38.26)	<0.001	≥15	403.20(399.28–407.12)	33.66(29.65–37.66)	<0.001
**Overall alcohol intake**				**Beer intake**			
Never drink	375.85(372.52–379.18)	Ref	NA	Never	388.28(385.19–391.37)	Ref	NA
Low	386.35(383.71–388.98)	10.50(6.99–14.00)		Low	386.49(382.70–390.28)	−1.79(−6.32–2.75)	0.441
Moderate	394.53(388.37–400.69)	18.68(13.09–24.26)		Moderate	395.74(390.06–401.42)	7.46(0.91–14.02)	0.026
High	401.44(396.75–406.14)	25.59(19.45–31.73)		High	402.65(396.57–408.73)	14.37(8.16–20.59)	<0.001
				**Spirits intake**			
				Never	381.59(377.69–385.49)	Ref	NA
				Low	391.65(388.47–394.83)	10.06(6.56–13.56)	<0.001
				Moderate	397.23(390.86–403.61)	15.64(9.29–22.00)	<0.001
				High	402.69(397.83–407.55)	21.10(14.83–27.37)	<0.001
				**Beer/spirits combination** ^*^			
				Never drink	374.90(371.26 −378.54)	Ref	NA
				Consumption of spirits with never beer intake	389.36(385.80–392.91)	14.45(9.25–19.66)	<0.001
				Both beer and spirits intake	398.40(393.38–403.43)	23.50(17.29–29.71)	<0.001

*BMI, body mass index, kg/m^2^; BFP, body fat percentage; VFI, vertical fat index; B: regression coefficient; CI: confidence interval; NA: not applicable*.

*Models 1 and 2 were adjusted for age, serum creatinine, personal annual income, residential areas, study sites, and smoking status. Overall alcohol intake categories, low: daily alcohol intake less than 40 g/day, moderate: 41–60 g/day, and high: >60 g/day. The grouping criteria of spirits intake is the same with overall alcohol intake. Beer intake categories, Low: no more than 20 bottles 1 year; moderate: no more than 40 bottles 1 year; and high: more than 40 bottles 1 year*.

* *The number of no spirits intake but only beer was limited, thus excluded from the analysis in the subgroup analysis*.

From never drinking to high alcohol consumption, the SUA levels increased positively (*p* < 0.001). Similar results were observed when distinguishing alcohol use into beer and spirits intake (*p* < 0.001). The high alcohol intake group had an average higher SUA level of 25.6 μmol/L than the never drink group. This effect was stronger in spirits intake, with an average higher SUA of 21.1 μmol/L than the never drink group (as reference), compared with 14.4 μmol/L in beer intake.

Excess adiposity was also positively associated with the SUA level. With elevated body fat distribution, the SUA level increased correspondingly. Compared with the nonobese group (under/normal weight), individuals with overweight and obesity had 34.03 and 64.36 μmol/L higher SUA levels, respectively (Model 1, Table 2). Likewise, subjects with higher BFP or VFI also had higher SUA levels (Table 2).

**Figure 1:**
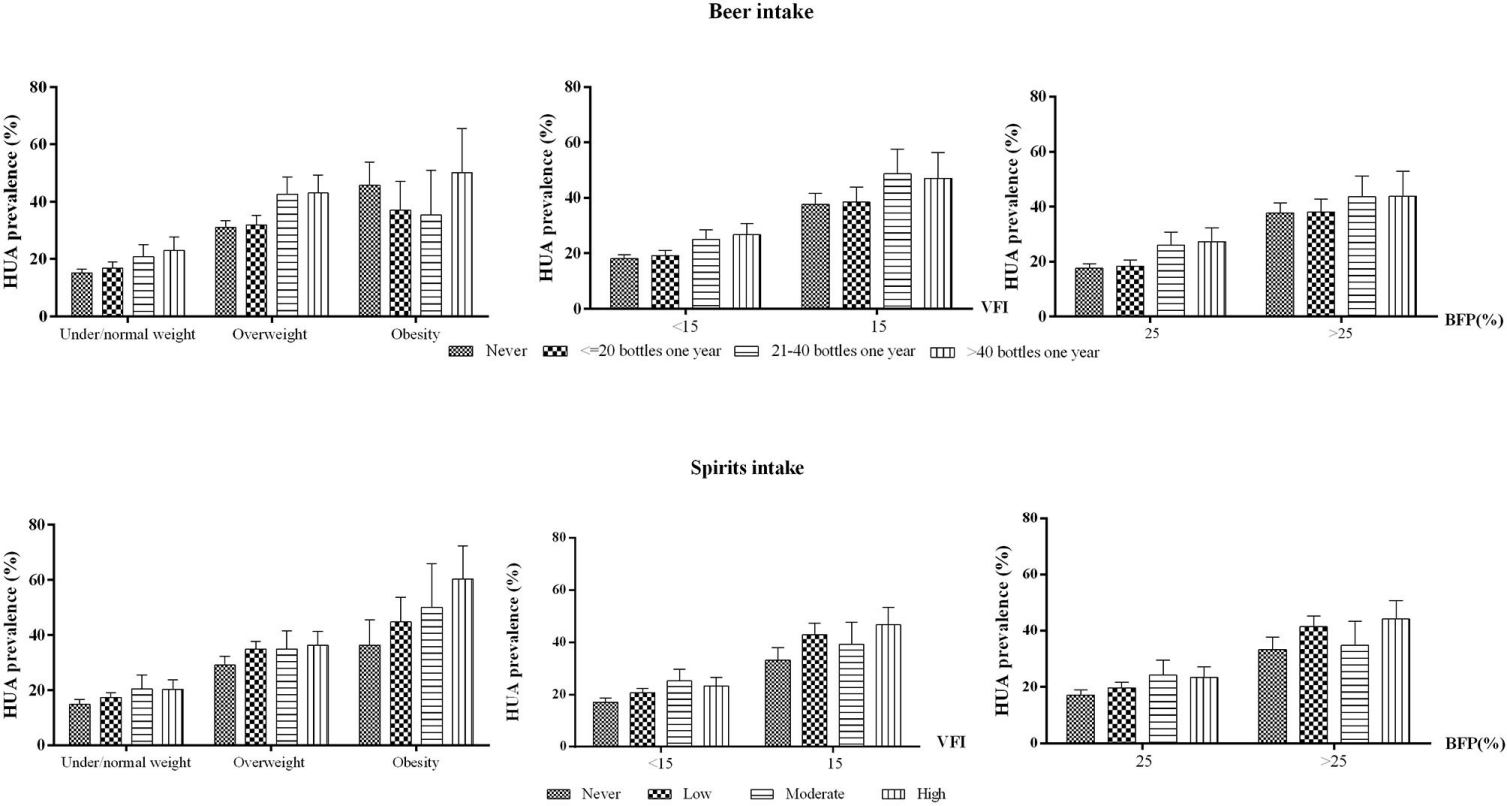
HUA prevalence in different excess adiposity and alcohol intake groups among men aged 20–80 in mainland China. HUA, hyperuricemia. BFP, body fat percentage. VFI, visceral fat index. Note: The prevalence was adjusted for age, residential areas, and study sites.

The prevalence of hyperuricemia among risk factor groups is shown in Figure 1. In general, the prevalence of HUA increased with the elevated body fat distribution measured by BMI, BFP, and VFI. Obese people who consumed beer more than 40 bottles 1 year had an HUA prevalence of 50.1% (95%CI: 34.7–65.5%), in comparison with the lowest HUA prevalence of 15.2% (95%CI: 13.9–16.5%) in people who never drink and were nonobese (*p* < 0.001). Other combinations of adiposity and alcohol intake had similar trends (Figure 1).

All excess adiposity indexes were found to be positively associated with the odds of HUA (Table 3), with *p*-values for trends <0.001. Either beer or spirits intake was associated with increased odds of HUA, and there were dose-response relationships between consumption levels and the risk of HUA (*p* < 0.001).

**Table 3:** The effect of excess adiposity and alcohol use on hyperuricemia (HUA) among male participants (*n* = 12,592).

	**Model 1**		**Model 2**		**Model 3**	
	**OR (95% CI)**	***P***	**OR (95% CI)**	***P***	**OR (95% CI)**	***P***
**BMI**						
Under/normal weight	Ref	NA	Ref	NA	Ref	NA
Overweight	2.38 (2.14–2.65)	<0.001	2.42 (2.17–2.69)	<0.001	2.33 (2.09–2.60)	<0.001
Obesity	4.43 (3.44–5.71)	<0.001	4.28 (3.57–5.14)	<0.001	4.24 (3.52–5.11)	<0.001
*P _*trend*_*	<0.001	<0.001	<0.001			
**BFP (%)**						
Q1	Ref	NA	Ref	NA	Ref	NA
Q2	1.03 (0.71–1.48)	0.891	1.66 (1.37–2.01)	<0.001	1.62 (1.36–1.93)	<0.001
Q3	1.78 (1.25–2.52)	0.001	2.90 (2.53–3.32)	<0.001	2.78 (2.43–3.18)	<0.001
Q4	2.88 (2.00–4.14)	<0.001	4.71 (3.92–5.66)	<0.001	4.48 (3.76–5.33)	<0.001
*P _*trend*_*	<0.001		<0.001		<0.001	
**VFI**						
<9	Ref	NA	Ref	NA	Ref	NA
9–14	2.34 (2.05–2.68)	<0.001	3.12 (2.75–3.54)	<0.001	2.96 (2.59–3.39)	<0.001
≥15	3.48 (3.02–4.01)	<0.001	6.12 (5.23–7.17)	<0.001	5.68 (4.80–6.71)	<0.001
*P _*trend*_*	<0.001		<0.001		<0.001	
**Beer intake**						
Never	Ref	NA	Ref	NA	Ref	NA
Low	1.14 (0.97–1.33)	0.104	0.97 (0.84–1.11)	0.619	0.96 (0.83–1.10)	0.532
Moderate	1.41 (1.08–1.83)	0.011	1.28 (1.07–1.54)	0.008	1.27 (1.05–1.54)	0.013
High	1.95 (1.52–2.49)	<0.001	1.42 (1.18–1.70)	<0.001	1.44 (1.20–1.73)	<0.001
*P _*trend*_*	<0.001		0.001		<0.001	
**Spirits intake**						
Never	Ref	NA	Ref	NA	Ref	NA
Low	1.56 (1.30–1.87)	<0.001	1.28 (1.12–1.45)	<0.001	1.26 (1.11–1.42)	<0.001
Moderate	1.82 (1.39–2.40)	<0.001	1.37 (1.11–1.69)	0.003	1.37 (1.13–1.65)	0.001
High	1.76 (1.35–2.29)	<0.001	1.35 (1.13–1.60)	<0.001	1.39 (1.19–1.63)	<0.001
*P _*trend*_*	<0.001		<0.001		<0.001	
Beer/spirits combination^*^						
Never drink	Ref	NA	Ref	NA	Ref	NA
Consumption of spirits with never beer intake	1.80 (1.43–2.28)	<0.001	1.42 (1.19–1.71)	<0.001	1.41 (1.19–1.68)	<0.001
Both beer and spirits intake	2.23 (1.67–2.97)	<0.001	1.86 (1.60–2.17)	<0.001	1.89 (1.62–2.20)	<0.001
*P _*trend*_*	<0.001		<0.001		<0.001	

*BMI, body mass index, kg/m^2^; BFP, body fat percentage; VFI, visceral fat index; OR, odds ratio; CI, confidence interval; NA, not applicable; Ref: reference*.

*Model 1 is the univariate analysis. Model 2 adjusted for age and study sites. Model 3 additionally adjusted for residential areas, income, smoking status, and creatinine level. Beer intake categories, low: no more than 20 bottles 1 year; moderate: no more than 40 bottles 1 year; and high: more than 40 bottles 1 year. Spirits intake categories, low: daily alcohol intake <40 g/day, moderate: 41–60 g/day, and high: >60 g/day. The grouping criteria of spirits intake are the same with an overall alcohol intake*.

* *The number of no spirits intake but only beer was limited, thus excluded from the analysis in the sup-group analysis. Q1–Q4 represented for the first to the fourth quartile of BFP*.

### The Joint Effect of Excess Adiposity and Alcohol Use on HUA

We demonstrated the prevalence of HUA and estimated the ORs for each risk factor and its combinations (Table 4). After the estimation of the joint effect of overall alcohol use with adiposity on HUA, we found that current drinking in the obese population (BMI > 25, BFP > 25, or VFI > 15) had the highest HUA prevalence and the highest ORs. Positive RERI, AP, and SI were observed among all the three adiposity indexes. For obese men who were presently drinking alcohol, the RERI was 0.65 (95%CI: 0.35–0.94), with an SI of 1.37 (95%CI: 1.12–1.67), indicating that the integration of alcohol use and overweight/obesity had a greater health effect than the sum of the independent effect of the two factors. By calculating AP, we could know that 19% (95%CI: 10–29%) of the combined risk of overweight/obesity and alcohol use were due to an additive interaction. Similar demonstrations could be drawn using BFP and VFI adiposity indexes. Interestingly, VFI seemed to have a greater additive interaction than the other two adiposity indexes, based on the larger values of its RERI, AP, and SI.

**Table 4:** The effect of excess adiposity and alcohol intake and their additive interaction on HUA among men aged 20–80 in mainland China.

	**Overall Alcohol Intake**	***n***	**% HUA**	**OR (95% CI)**	***P***	**RERI (95% CI)**	**AP (95% CI)**	**SI (95% CI)**
**BMI category**								
Under/normal weight	Never drink	2,004	12.23	Ref	NA	Ref	Ref	Ref
	Current drink	5,065	18.30	1.31 (1.10–1.55)	0.002			
Overweight/obesity	Never drink	1,119	25.16	2.44 (2.01–2.97)	<0.001			
	Current drink	4,135	36.47	3.40 (2.88–4.00)	<0.001	0.65 (0.35–0.94)	0.19 (0.10–0.29)	1.37 (1.12–1.67)
**BFP (%)**								
≤ 25	Never drink	2,306	13.27	Ref	NA	Ref	Ref	Ref
	Current drink	5,690	20.42	1.33 (1.17–1.52)	<0.001			
>25	Never	588	27.89	2.36 (1.94–2.87)	<0.001			
	Current drink	2,099	40.35	3.40 (2.89–4.01)	<0.001	0.71 (0.30–1.12)	0.21 (0.10–0.32)	1.420 (1.13–1.79)
**VFI**								
≤ 15	Never drink	2,431	15.30	Ref	NA	Ref	Ref	Ref
	Current drink	6,997	23.55	1.31 (1.17–1.46)	<0.001			
>15	Never drink	646	22.29	1.97 (1.66–2.33)	<0.001			
	Current drink	2,110	36.45	3.12 (2.69–3.62)	<0.001	0.85 (0.41–1.28)	0.27 (0.15–0.39)	1.66 (1.25–2.21)
**Beer intake**								
**BMI category**								
Under/normal weight	Never/low	6,791	16.04	Ref	NA	Ref	Ref	Ref
	Moderate/high	379	25.86	1.41 (1.11–1.79)	0.005			
Overweight/obesity	Never/low	4,941	33.29	2.54 (2.31–2.79)	<0.001			
	Moderate/high	356	46.07	3.53 (2.76–4.50)	<0.001	0.58 (−0.41–1.57)	0.17 (−0.08–0.41)	1.30 (0.85–1.97)
**BFP (%)**								
≤ 25	Never/low	7,709	17.77	Ref	NA	Ref	Ref	Ref
	Moderate/high	396	29.55	1.68 (1.38–2.04)	<0.001			
>25	Never/low	2,578	37.39	2.61 (2.31–2.94)	<0.001			
	Moderate/high	141	44.68	2.88 (1.78–4.65)	<0.001	−0.40 (−1.81–1.00)	−0.14 (−0.69–0.41)	0.82 (0.39–1.72)
**VFI**								
≤ 15	Never/low	8,963	20.63	Ref	NA	Ref	Ref	Ref
	Moderate/high	586	33.45	1.56 (1.33–1.83)	<0.001			
>15	Never/low	2,645	32.55	2.30 (2.05–2.57)	<0.001			
	Moderate/high	144	44.44	3.12 (2.14–4.27)	<0.001	0.17(yy−0.90–1.25)	0.06(y−0.28–0.40)	1.09 (0.64–1.87)
**Spirits intake**								
**BMI category**								
Under/normal weight	Never drink	2,611	13.67	Ref	NA	Ref	Ref	Ref
	Current drink	4,048	18.75	1.26 (1.05–1.51)	<0.001			
Overweight/obesity	Never drink	1,407	27.93	2.50 (2.12–2.96)	<0.001			
	Current drink	3,574	36.71	3.20 (2.71–3.79)	<0.001	0.45 (0.08–0.81)	0.14 (0.03–0.25)	1.25 (1.02–1.54)
**BFP (%)**								
≤ 25	Never drink	2,941	15.06	Ref	NA	Ref		
	Current drink	4,610	20.74	1.22 (1.03–1.44)	0.023			
>25	Never	731	30.10	2.31 (1.90–2.80)	<0.001			
	Current drink	1,847	40.99	3.13 (2.63–3.73)	<0.001	0.61 (0.26–0.97)	0.20 (0.09–0.31)	1.40 (1.11–1.77)
**VFI**								
≤ 15	Never drink	3,219	17.27	Ref	NA	Ref		
	Current drink	5,667	24.16	1.27 (1.11–1.45)	<0.001			
>15	Never drink	759	24.51	2.06 (1.77–2.39)	<0.001			
	Current drink	1,877	36.49	2.92 (2.49–3.44)	<0.001	0.59 (0.12–1.07)	0.20 (0.06–0.35)	1.45 (1.07–1.95)

*BMI, body mass index, kg/m^2^; BFP, body fat percentage, %; VFI, visceral fat index; OR, odds ratio; CI, confidence interval; RERI, relative excess risk due to interaction; AP, attributable proportion due to interaction; SI, synergy index; NA, not applicable; Ref, reference*.

*Beer intake categories, low: no more than 20 bottles 1 year; moderate: no more than 40 bottles 1 year; and high: more than 40 bottles 1 year. The multivariate regression models were adjusted for age, personal annual income, residential areas, study sites, and serum creatinine level. RERI and AP larger than 0 and SI large than 1 indicate a positive additive interaction effect*.

We distinguished alcohol use into beer and spirits to see if there was a difference between alcohol beverage kinds in the interaction estimates. The results showed that, although people who were obese with a moderate/high intake of beer had the highest OR than the reference group, there was no additive interaction (Table 4). On the contrary, spirits intake had both independent and additive interaction effects. Obese people who were presently drinking spirits had an OR of 3.20 (the never drink group as a reference), and the RERI was larger than 0, 0.45 with 95% CI of 0.08–0.81, the SI was 1.25 (95%CI: 1.02–1.54). Around 14% (3–25%) of the combined risk can be attributed to the additive interaction. The results were found to be consistent among different adiposity indexes.

## Discussion

To the best of our knowledge, this is the first study aimed to understand an additive interaction effect between alcohol use and excess adiposity on HUA. Using data from the CNHS, we demonstrated that body composition and alcohol use had an additive interaction effect on HUA, after adjusting for potential confounders. Furthermore, spirits intake, but not beer, had an interaction on the additive scale with adiposity in the study population.

The relationship between adiposity and SUA has been well-explored. In a large survey of 310,577 Japanese middle-aged adults, obese participants were found to have a 1.33–3.76-fold higher risk of HUA in comparison with their nonobese counterparts (29). Other cross-sectional studies also indicated positive associations between BMI and HUA (30, 31). BMI has been acknowledged to have a causal and independent effect on elevating the SUA level (5, 30, 32). However, BMI does not differentiate fat-free mass from adipose tissue (33) and may have a low sensitivity for predicting BFP (34). Therefore, we used the two other adiposity indexes, BFP and VFI, as alternative indicators of excess adiposity. In a Chinese hypertension registry study, BFP was found to be positively associated with an increased risk of HUA among hypertensive patients (35). BFP was also found to be associated with cardiometabolic disorders in a variety of populations (36, 37). Excess body fat increases morbidity, with excess visceral fat being an important factor that triggers pathologies among cardiometabolic risk factors (38–41). Yamada et al. reported that visceral adiposity was independently associated with HUA in a Japanese population (42). The mechanisms underlying the relationship between visceral adiposity and HUA are not fully understood. The cause of HUA is known to be an overproduction or a reduction in renal or extrarenal excretion of uric acid. Thus, the possible mechanism may be the reduction of renal excretion of SUA due to hyperinsulinemia through adiposity (42, 43).

The measure of BMI could give both healthcare providers and patients quick feedback on the risk estimation due to its low cost, simple operation, and availability. BFP and VFI measurements enable the assessment of fat mass and its distribution. The use of different excess adiposity indexes in our study allows for more comprehensive perspectives on the exploration of HUA risk factors.

Another well-established modifiable risk factor of HUA is alcohol intake. For two prospective cohort studies in Japanese men, alcohol intake was found to be associated with the incidence of HUA (44, 45). Data from NHANES-III also indicated that alcohol beverage consumption is associated with elevated SUA and HUA (5), and beer conferred a larger increase than liquor, whereas wine drinking did not increase SUA (12). Similarly, in two cross-sectional surveys of Caucasian adults, and in the CARDIA cohort among US young adults with a 20-year follow-up, the consumption of beer and spirits, but not wine, was related with elevated SUA (11, 46). Our study also implied that the intake of beer and spirits was associated with elevated SUA and HUA.

A few previous studies explored HUA risk factors, including both alcohol use and obesity, but a few investigated their interaction effect. In a Japanese population, on the multiplicative scale, no interaction effect on HUA was observed (7). Interaction on the additive scale is more likely to reflect the biological interaction, and interaction measurement between excess adiposity and alcohol use enables the estimation of an excess risk due to both exposures. Our study revealed that both exposures of excess adiposity and alcohol drink had an additive interaction effect on the odds of HUA, which indicated an excess risk than the sum of the two risk factors. Public health intervention strategies and resource location should be paid more attention toward the population with both exposures. Furthermore, when we distinguished alcohol beverages into spirits and beer, the results showed that an additive interaction was mainly attributed to spirits intake but not beer. One possible reason, which is also one of the limitations of the study, is that there was a limited sample size in the subgroup of beer intake. For instance, never or low consumption of beer accounted for over 80% of the study population, whereas over 60% of participants had an average alcohol intake level of at least 40 g/day in the past 1 year. In a different perspective, given that there was limited research focusing on an additive interaction between excess adiposity and beer intake, further investigation is needed to fully understand the mechanism.

There are several limitations to this study. First, conclusions on causal relationships cannot be drawn due to the nature of a cross-sectional design. Second, we did not collect information on genetic and dietary factors, and other unmeasured confounders may exist. Nonetheless, evidence suggested that diet and other modifiable risk factors, such as exercise, influenced the risk of HUA and gout through modifying BMI (47), thus making it non-reasonable to adjust dietary factors as confounders in the multivariate analysis based on Rothman's recommendation (14). Third, we did not collect information on urate lowering drug use, thus possibly leading to a nondifferential measurement error, and biased our estimates toward the null, resulting in a more conservative assessment. Moreover, the prevalence of HUA and health profiles on adiposity and alcohol intake may vary during the survey year, thus inducing a misclassification bias. Nevertheless, as our main purpose is to estimate an interaction effect of adiposity and alcohol intake on HUA, not the prevalence of these factors, the study conclusion may not be substantially influenced.

The strengths of this study are the analyses conducted within a large, ethnically homogeneous sample. A rigid design and the use of standardized measurement throughout the survey place it particularly well for the estimation of an additive interaction effect among areas. Moreover, we use the three indexes, namely BMI, BFP, and VFI, to measure excess adiposity, thus making a more comprehensive exploration of health estimates. Distinguishing different alcoholic beverages allow for more precise evidence of health risk estimates. Taken together, our findings embolden the effect of modifiable risk factors, i.e., excess adiposity and alcohol use, on elevated SUA, particularly in the way of indicating a joint effect of the two important lifestyle factors on a biological interactive perspective.

In summary, excess adiposity and alcohol use were found to be positively associated with the prevalence of HUA and SUA levels. Overweight/obesity, high BFP, and high VFI were all found to have an interaction effect with spirits intake, but not beer, on HUA on the additive scale. These findings further strengthen public health recommendations to identify high priority populations and initiate more targeted health interventions.

## Data Availability Statement

The original contributions presented in the study are included in the article/supplementary material, further inquiries can be directed to the corresponding author.

## Ethics Statement

The studies involving human participants were reviewed and approved by the Bioethical Committee of Institute of Basic Medical Sciences, Chinese Academy of Medical Sciences. The participants provided their written informed consent to participate in this study.

## Author Contributions

HH and GS: conceptualization, data curation, and funding acquisition. HH: methodology, software, and formal analysis and writing—original draft preparation. HH, GS, and LPan: validation, project administration, and supervision. LPan, XR, DW, JD, ZC, HW, XW, FL, LPa, XP, YW, CY, GS, and HH: investigation. GS, HH, XR, DW, JD, ZC, HW, XW, FL, LPa, XP, YW, and CY: resources. GS: writing—review and editing. HH, YW, and CY: visualization. All authors read and approved the final manuscript.

## Funding

This work was funded by the National Key R&D Program of China (Grant No. 2016YFC0900600/2016YFC0900601), the Key Basic Research Program of the Ministry of Science and Technology of China (Grant No. 2013FY114100), the National Natural Science Foundation of China (Grant No. 82003531), and Science & Technology Fundamental Resources Investigation Program (Grant No. 2017FY101100/2017FY101101).

## Conflict of Interest

The authors declare that the research was conducted in the absence of any commercial or financial relationships that could be construed as a potential conflict of interest.

## Publisher's Note

All claims expressed in this article are solely those of the authors and do not necessarily represent those of their affiliated organizations, or those of the publisher, the editors and the reviewers. Any product that may be evaluated in this article, or claim that may be made by its manufacturer, is not guaranteed or endorsed by the publisher.
